# Fractal Dimension Analysis of Mandibular Trabecular Bone in Patients Receiving Antiresorptive Therapy for Osteoporosis and Oncologic Conditions

**DOI:** 10.3390/diagnostics15060748

**Published:** 2025-03-17

**Authors:** Mehmet Altay Sevimay, Müjde Gürsu, Muhammed Abdullah Çege, Dilek Aynur Çankal, Zühre Akarslan, Sedat Çetiner

**Affiliations:** 1 Department of Oral and Maxillofacial Surgery, Faculty of Dentistry, Gazi University, 06490 Ankara, Turkey; mujdegursu@hotmail.com (M.G.); ma.cege26@gmail.com (M.A.Ç.); dugar@gazi.edu.tr (D.A.Ç.); scetiner@gazi.edu.tr (S.Ç.); 2 Department of Oral and Maxillofacial Radiology, Faculty of Dentistry, Gazi University, 06490 Ankara, Turkey; zakarslan@gazi.edu.tr

**Keywords:** bone density conservation agents, fractals, osteoporosis, bisphosphonates, denosumab

## Abstract

**Objectives**: This study aimed to assess the effects of antiresorptive drugs on mandibular trabecular bone structure in patients with osteoporosis and those receiving antiresorptive therapy for oncologic conditions using fractal dimension (FD) analysis of panoramic radiographs. Additionally, it investigated the influences of age, gender, drug type, administration route, and treatment duration on mandibular trabecular bone structure. **Methods:** This retrospective cross-sectional study included 73 patients categorized into the following three groups: 23 osteoporosis patients, 25 oncologic patients, and 25 systemically healthy controls. FD analysis was conducted on panoramic radiographs to assess trabecular bone complexity in the following three standardized regions of interest: the mandibular condyle, angle, and molar region. Statistical analyses compared the groups and evaluated the associations between FD values and demographic and clinical parameters. **Results**: Osteoporosis patients exhibited significantly lower FD values in the molar region than controls (*p* < 0.05). In oncologic patients, the FD values in the condyle region were significantly higher in those receiving denosumab than in those treated with intravenous zoledronic acid (*p* < 0.05), and in those who had undergone antiresorptive therapy for ≥6 years than in those treated for 1–5 years (*p* < 0.05). A significant negative correlation was found between age and the FD values of the mandibular angle in osteoporosis patients (*p* < 0.05); no such association was observed in oncologic patients or controls. **Conclusions**: Long-term antiresorptive therapy may induce structural alterations in mandibular trabecular bone structure in patients with osteoporosis and oncologic diseases. FD analysis is a non-invasive and objective tool for clinically assessing such drug-induced skeletal changes. However, further large-scale, prospective studies are necessary to confirm these findings and shed light on their clinical significance.

## 1. Introduction

Antiresorptive drugs are therapeutic agents widely used to treat osteoporosis and metabolic bone diseases and manage skeletal complications in oncologic patients. In osteoporosis cases, these drugs aim to reduce fracture risk by preserving bone mineral density, whereas, in oncologic patients, they are utilized to alleviate bone pain, improve quality of life, and prevent skeletal complications associated with conditions such as hypercalcemia in multiple myeloma and bone metastases from breast, prostate, and lung cancers. In both cases, these agents play a crucial role in maintaining skeletal integrity by inhibiting bone resorption. Among antiresorptive drugs, bisphosphonates and denosumab are the most commonly used agents [1,2]. 

Bisphosphonates primarily inhibit osteoclastic activity by binding to hydroxyapatite crystals in actively remodeling bone. However, they have also been reported to suppress osteoblastic activity and impair the growth and healing of mucosal epithelial cells [3]. Meanwhile, denosumab reduces bone resorption by inhibiting osteoclast function [4]. Despite their therapeutic benefits, both agents have been associated with severe adverse effects, particularly medication-related osteonecrosis of the jaw (MRONJ). MRONJ is characterized by impaired healing and progressive bone destruction due to compromised blood circulation [5].

While the effects of antiresorptive drugs on long bones and vertebrae have been extensively studied [6,7], their impact on craniofacial structures, particularly the mandible, remains less understood. Given the mandible’s unique remodeling pattern and direct involvement in various dental procedures, evaluating how these drugs affect mandibular trabecular bone is of great clinical significance. Understanding these effects is crucial for optimizing dental treatment planning, implant placement, and assessing osteonecrosis risk in patients undergoing antiresorptive therapy [8].

Oral antiresorptive drugs are typically prescribed for the treatment of osteopenia or osteoporosis, whereas intravenous (IV) antiresorptive drugs are primarily used in the management of malignancies that metastasize to bone, such as breast, prostate, and lung cancers. This distinction arises because IV bisphosphonates have a higher bioavailability than oral bisphosphonates [9]. Despite their therapeutic benefits, these agents are associated with serious adverse effects, particularly jaw osteonecrosis, which is characterized by progressive bone destruction and is linked to impaired blood circulation in the jawbone [3]. This adverse effect has been reported to occur particularly with nitrogen-containing bisphosphonates or following IV administration and is associated with the cumulative dose of the antiresorptive drug [10]. Additionally, in patients treated with bisphosphonates, the risk of developing jaw osteonecrosis has been documented to increase after 12 months of IV therapy and typically after 3 years or more of oral administration [11].

Marx et al. first reported in 2003 that jaw osteonecrosis could develop in patients using bisphosphonates, defining the condition as “bisphosphonate-related osteonecrosis of the jaw” (BRONJ) [12]. However, further studies demonstrated that other medications could also induce jaw osteonecrosis. Accordingly, in 2014, the American Association of Oral and Maxillofacial Surgeons (AAOMS) updated the terminology, redefining the condition as MRONJ to emphasize that osteonecrosis is not exclusively associated with bisphosphonates [13].

In patients receiving antiresorptive therapy who present with exposed bone, a diagnosis of MRONJ can be established. However, approximately 30% of MRONJ cases occur without bone exposure, highlighting the critical role of radiographic evaluation, particularly in the early detection of the condition [14]. Although radiographic findings for early-stage MRONJ are not highly specific, several characteristic features have been described, including thickening of the lamina dura, osteolysis, alveolar bone loss unrelated to periodontal disease, and the absence of remodeling in extraction sockets [15].

Advancements in digital technology and imaging analysis techniques have facilitated the quantitative assessment of alveolar bone changes [16]. Dental panoramic radiographs are widely regarded as a vital diagnostic modality in dentistry owing to their minimal radiation exposure, affordability, and cost-effectiveness [17].

Fractal dimension (FD) analysis is a mathematical technique that enables the quantitative assessment of complex structures, including trabecular bone [18]. This structural complexity is represented by a single numerical value computed through a specialized algorithm [19]. Given the self-similar and branching nature of trabecular bone, which exhibits fractal properties, FD analysis is a valuable tool for characterizing its structure [20]. The application of this method to radiographs of trabecular bone allows for a reflection of the bone microarchitecture and serves as a non-invasive approach for detecting and quantifying bone alterations [21]. FD analysis is widely utilized in dentistry to assess trabecular bone structure in various conditions, including bone remodeling, periapical bone changes, apical healing, and osteoporosis [19]. Notably, higher FD values correlate with an increased structural complexity within the bone architecture [22]. Dental panoramic radiographs have been successfully applied in conjunction with FD analysis and radio morphometric measurements to assess the influence of systemic diseases and medications on the mandible [23].

A limited number of studies have utilized FD analysis to investigate changes in bone structure among patients with MRONJ and explore potential correlations between the disease and radiographic features [16,17,24]. Existing research primarily focuses on the FD analysis of mandibular trabecular bone, comparing osteoporosis patients receiving bisphosphonates with control groups to assess bone structural alterations. To the best of our knowledge, no study has yet evaluated and compared trabecular bone changes in the mandible of both oncologic and osteoporosis patients receiving antiresorptive therapy using FD analysis. Thus, the present study aimed to assess potential changes in the mandibular trabecular bone structure associated with antiresorptive drug use in osteoporosis and oncologic patients utilizing digital panoramic radiography. Furthermore, the study sought to evaluate the impacts of key parameters, including the type of antiresorptive drug, route of administration, treatment duration, age, and gender, on trabecular bone alterations within these patient groups.

This study tests the hypothesis that the FD values of the mandibular trabecular bone do not differ significantly between osteoporotic and oncologic patients receiving antiresorptive therapy and systemically healthy controls.

## 2. Materials and Methods

This study was approved by the Gazi University Ethics Committee (Decision No. E.1130252 on 31 December 2024) and conducted following the ethical principles outlined in the Declaration of Helsinki.

### 2.1. Patient Selection and Data Collection

This cross-sectional study retrospectively analyzed standardized panoramic radiographic images of patients who presented to the Faculty of Dentistry, Gazi University, between January 2021 and January 2025 and were receiving antiresorptive medication (bisphosphonates/denosumab) for osteoporosis or oncologic conditions. Demographic data and comprehensive medical history records for all patients, including the type of antiresorptive drug, duration of use, and route of administration, were accessed through the university’s patient database.

The inclusion criteria for this study consisted of adult patients who presented to our clinic and had been using antiresorptive drugs for at least one year to treat osteoporosis or oncologic conditions. The patients were categorized into groups based on their clinical presentation and history of antiresorptive drug use, with no restrictions regarding age or gender. In both the osteoporosis and oncologic patient groups, only those who were actively undergoing treatment were included. The control group comprised systemically healthy individuals with no history of medication use that could affect bone metabolism. These individuals were selected to match the study groups in terms of age and gender distribution.

The exclusion criteria included patients with metabolic bone diseases other than osteoporosis, oncologic patients who had undergone head and neck radiotherapy, individuals with a history of jaw osteonecrosis, and those using antiresorptive drugs for reasons other than osteoporosis or cancer. To minimize confounding factors, smokers were also excluded. To ensure the accuracy of radiographic assessments, patients with poor-quality panoramic radiographs—such as those with artifacts, pathological lesions, horizontal or vertical distortion, or overlapping anatomical structures that prevented proper evaluation—were also excluded. Radiographic analyses were conducted only on images where the mandibular cortical bone, condyle, and inferior border were clearly visible and traceable without artifacts.

The study population was divided into the following three groups: 23 patients (22 females and 1 male) using antiresorptive drugs for osteoporosis; 25 patients (13 males and 12 females) receiving antiresorptive medication for the treatment of various types of cancer, including breast cancer (*n* = 8), lung cancer (*n* = 5), prostate cancer (*n* = 5), and other malignant tumors (*n* = 7); and 25 systemically healthy individuals (13 males and 12 females) with no history of medication use. The distribution of antiresorptive drug use among the patient groups was as follows: in the osteoporosis group, 11 patients were receiving IV zoledronic acid, while 12 patients were taking oral alendronic acid. In the oncologic patient group, 17 patients were treated with IV zoledronic acid, and 8 patients were receiving denosumab. Regarding the duration of antiresorptive drug use, in the oncologic patient group, 19 patients had been receiving treatment for 1–5 years, while 6 patients had been on therapy for 6 years or more. In the osteoporosis group, 13 patients had been treated for 1–5 years, whereas 10 patients had used the medication for 6 years or longer (Table 1).

### 2.2. Fractal Dimension Analysis

Digital panoramic radiographs were obtained using a Sirona Dental Systems (Bensheim, Germany) machine operating at 66 kVp and 8 mA, with a 0.5 mm focal spot and a 14-second exposure time in a standard positioning protocol. All radiographs were acquired by an experienced radiology technician. The panoramic images were stored in JPEG format with a resolution of 2440 × 1292 pixels at 96 dpi and a 24-bit color depth. Radiographic assessments were performed on a 15.6-inch laptop (Excalibur G770, Casper, Istanbul, Turkey) with a 1920 × 1080 resolution and 32-bit color depth in a quiet room under subdued lighting conditions.

**Figure 1:**
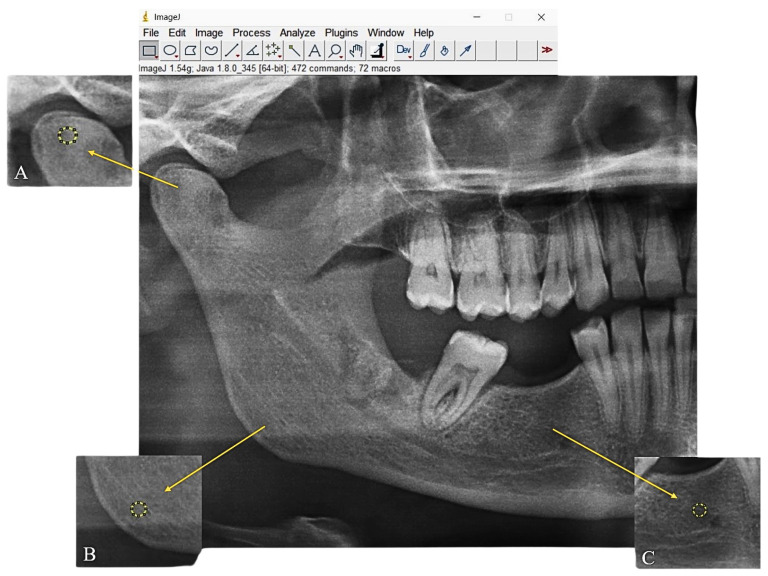
Panoramic radiograph displaying the selected regions of interest (ROIs). (**A**) ROI-1: mandibular condyle, (**B**) ROI-2: center of mandibular angle, and (**C**) ROI-3: mandibular molar area.

The panoramic radiographs from all three groups were converted into Tagged Image File Format (TIFF) due to their high resolution. To minimize potential misinterpretations caused by inflammatory changes, periapical and periodontal regions were excluded from the analysis. Regions of interest (ROIs) were manually selected from standardized anatomical locations to ensure consistency. For each patient, the following three standardized ROIs were identified:

- ROI-1: A 30 × 30-pixel circular region located at the geometric center of the right mandibular condyle.
- ROI-2: A 30 × 30-pixel circular region positioned at the geometric center of the right mandibular angle.
- ROI-3: A 30 × 30-pixel circular region located in the right mandibular molar area (Figure 1).

**Figure 2:**
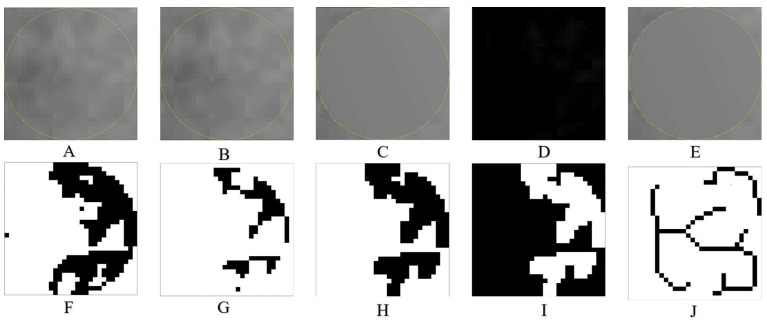
Image processing steps for fractal analysis. (**A**) Cropped grayscale image, (**B**) duplicated image, (**C**) Gaussian-blurred image to reduce noise, (**D**) subtracted image to enhance edges, (**E**) image with added intensity (128) for normalization, (**F**) binarized image, (**G**) eroded image for noise reduction, (**H**) dilated image to enhance structures, (**I**) inverted binary image, and (**J**) skeletonized image for structural simplification.

FD analysis was performed using the box-counting method, following the image processing methodology proposed by White and Rudolph [22]. The procedures were performed using ImageJ 1.8v software (National Institutes of Health, Bethesda, MD, USA). ROIs were defined at a size of 30 × 30 pixels, then cropped and duplicated. To minimize brightness variations resulting from overlapping soft tissues and differences in bone thickness, a Gaussian blur filter was applied. The processed image was then subtracted from the original to enhance structural contrast. To differentiate trabecular structures from bone marrow spaces, a gray value of 128 was added to each pixel location. Subsequently, a series of image processing steps, including binarization, erosion, dilation, inversion, and skeletonization, was applied to prepare the image for FD analysis (Figure 2).

All data were anonymized, and analyses were performed blinded to patient medical information. The evaluations were conducted by a radiologist with 24 years of experience in oral and maxillofacial radiology (Z.A.) and an oral and maxillofacial surgeon with 5 years of experience (M.A.Ç.), both trained in FD analysis. Interobserver agreement was assessed through a double-blinded evaluation, and a statistical analysis of measurement consistency was conducted. 

### 2.3. Statistical Analysis

The sample size was determined using G*Power 3.1.9.2 (Heinrich-Heine-Universität Düsseldorf, Germany). Power analysis indicated that a minimum of 22 individuals per group was required to achieve an effect size of 0.40, [25] an error margin of 0.05, a 95% confidence interval, and a population representation power of 0.80. A total of 73 patients were included in the study. 

In this study, data were analyzed using the Statistical Package for Social Sciences (SPSS) software, Windows version 27 (SPSS Inc., Chicago, IL, USA). Descriptive statistics, including frequency, percentage, mean, standard deviation, minimum, maximum, and median values, are presented. Associations between categorical variables across groups were evaluated using the chi-square test. When more than 20% of the expected cell counts were below 5, Fisher’s exact chi-square test was utilized. The Shapiro–Wilk test was employed to assess the normality of data distribution, while Levene’s test was used to evaluate the homogeneity of variances. If the normality assumption was satisfied, the independent samples t-test was applied for comparisons between two independent groups, whereas the paired samples t-test was used for comparisons between related groups. When normality was not achieved, non-parametric tests such as the Mann–Whitney U test and Wilcoxon signed-rank test were applied. For analyses involving three or more independent groups, one-way analysis of variance (ANOVA) was used when the data followed a normal distribution, while the Kruskal–Wallis test was applied for non-normally distributed data. Post hoc Bonferroni adjustments were made to identify specific group differences. To determine the reliability and consistency of measurements, both the Spearman correlation coefficient and intraclass correlation coefficient (ICC) were calculated. A significance level (α) of 0.05 was established for all statistical procedures.  

## 3. Results

Systemic interobserver reliability was assessed using the Spearman correlation coefficient and the ICC for FD measurements in the mandibular condyle, angle, and molar regions. The Spearman correlation coefficient (r) was high and statistically significant (*p* < 0.05) for all measurements. Similarly, the ICC values indicated a strong level of consistency and reliability. The ICC values for the mandibular condyle (0.906) and mandibular angle (0.986) regions demonstrated an excellent reliability, whereas the molar region (0.773) exhibited a relatively lower ICC value; however, it remained within the range of a high reliability. 

The study included 73 participants, divided into the following three groups: 25 oncologic patients, 23 osteoporosis patients, and 25 controls. The mean ages were 59.16 ± 11.32 years (range 41–87 years) for both the oncologic patient and control groups and 68.78 ± 9.6 years (range 49–90 years) for the osteoporosis group. The analysis revealed no statistically significant correlation between age and FD measurements in the mandibular condyle, angle, and molar regions for both the oncologic patient and control groups (*p* > 0.05). However, in the osteoporosis group, a significant negative correlation was found between age and FD measurements in the mandibular angle region (r = −0.557, *p* = 0.006), indicating that the FD values in this region decreased with an increasing age. No significant correlations were observed in other regions within the osteoporosis group (*p* > 0.05); see Table 2.

The effect of gender on the FD values in the ROIs was evaluated across the study and control groups. No statistically significant differences were observed between female and male participants in the mandibular condyle, angle, and molar regions within the oncologic patient, osteoporosis, and control groups (*p* > 0.05); see Table 3.

A comparison of the mean FD values in the ROIs between the study and control groups revealed no statistically significant differences in the mandibular condyle (X^2^ = 0.556, *p* = 0.757) and angle (X^2^ = 2.846, *p* = 0.241) regions. However, a statistically significant difference was detected in the mandibular molar region (X^2^ = 10.063, *p* = 0.007). Specifically, osteoporosis patients exhibited significantly lower fractal measurements in the mandibular molar region (1.26 ± 0.18) compared to the control group (1.32 ± 0.07, *p* < 0.05); see Table 4.

The impacts of antiresorptive drug type and administration route on the FD values were analyzed within the study groups and across the ROIs to assess potential variations. In the oncologic patient group, the FD measurements in the mandibular condyle region were significantly higher in individuals receiving denosumab compared to those receiving IV zoledronic acid (t = 2.791, *p* = 0.010). However, no significant differences were observed in the mandibular angle and molar regions based on drug type (*p* > 0.05). Similarly, in the osteoporosis group, the FD values did not differ significantly between individuals receiving IV zoledronic acid and those using oral alendronic acid in the mandibular condyle, angle, or molar regions (Table 5). Additionally, in the oncologic patient group, individuals who had been using antiresorptive medication for 6 years or longer exhibited significantly higher FD measurements in the mandibular condyle compared to those who had been using the medication for 1–5 years (Z = −1.972, *p* = 0.049). However, no significant differences were found in the mandibular angle and molar regions based on treatment duration (*p* > 0.05). Similarly, in the osteoporosis group, FD values did not show any statistically significant differences between individuals with 1–5 years of medication use and those with 6 or more years of use in the mandibular condyle, angle, or molar regions (*p* > 0.05); see Table 6.

## 4. Discussion

Bisphosphonates and denosumab, both antiresorptive agents, are commonly used to treat various bone diseases, including osteoporosis, metastatic bone disease, and malignancy-associated hypercalcemia [26]. Although both drugs effectively reduce bone resorption, they exhibit distinct pharmacological properties and different effects on bone tissues. Bisphosphonates can be administered orally or intravenously, accumulate in bone without being metabolized, and have a long half-life, persisting in bone tissue for months to years. In contrast, denosumab, a monoclonal antibody administered via subcutaneous injection, is metabolized in the body, is not stored in bone, and has a shorter half-life of several weeks [27]. These pharmacokinetic differences lead to varying impacts on jawbones. Bisphosphonates are known to accumulate primarily in trabecular bone, increasing bone density in these regions, while denosumab has a more pronounced effect on cortical bone, with a limited influence on trabecular bone [28].

The risk of MRONJ in patients receiving bisphosphonate therapy necessitates a cautious approach during dental treatment [10,13]. Preventive strategies should be prioritized and interventions should be tailored to the patient’s risk level [29]. For low-risk patients, routine dental procedures such as restorations, endodontic treatments, and periodontal care can be performed with minimal modifications. However, invasive procedures, including extractions, implant placement, and periodontal surgeries should be carefully planned to account for the increased risk of MRONJ. When extractions or surgical interventions are necessary, atraumatic techniques should be employed, primary closure should be attempted, and prophylactic antibiotics should be considered to minimize complications [30]. Long-term follow-up and good oral hygiene are essential for reducing complications in these patients. Educating them about potential risks and emphasizing the importance of routine dental check-ups can help to prevent the need for invasive procedures and lower the likelihood of developing osteonecrosis [13].

In this context, this study aimed to analyze mandibular trabecular bone structure using an FD analysis of panoramic radiographs of patients using bisphosphonates or denosumab due to osteoporosis or oncologic conditions and compare the findings with those from a healthy control group.

Our results indicate that antiresorptive therapy has a significant impact on mandibular trabecular bone structure, with notable differences in the FD values among osteoporotic, oncologic, and control groups. Additionally, factors such as drug type, administration route, and treatment duration were found to influence bone microarchitecture. Based on these findings, the null hypothesis can be rejected, as long-term antiresorptive therapy appears to induce structural changes in the mandibular trabecular bone, supporting the notion that these treatments affect bone microarchitecture.

FD analysis is a mathematical method used to evaluate the structural complexity of bone tissue, playing a crucial role in the assessment of dynamic structures such as trabecular bone. FD, calculated from two-dimensional radiographic images, enables the identification of changes in bone architecture and density [31]. Higher FD values indicate a more complex, denser, and less porous bone structure [20], whereas lower FD values have been associated with bone loss and resorption processes [17]. Among the various methods available for FD calculation, the box-counting method is one of the most widely used approaches and was employed in the present study to assess mandibular trabecular architecture [31].

In dentistry, FD analysis has been utilized for various clinical applications, including the assessment of bone quality before implant placement, the detection of bone loss due to periodontal diseases, and the monitoring of bone healing following orthognathic surgery. Studies in periodontology have demonstrated that patients with moderate to severe periodontitis present significantly lower FD values than healthy individuals [32]. Furthermore, an increase in FD values has been observed in the periapical region following endodontic treatment [33], along with a progressive increase in FD values observed during the bone healing process after orthognathic surgery [34]. Additionally, comparative analyses before and after implant loading have revealed a significant increase in FD values 3 months after implant placement [35].

The changes induced by antiresorptive drugs in the jawbone are reported to depend on factors such as treatment duration, administration route, dosage, and drug type. Additionally, clinical studies have shown that these agents enhance bone mineral density [36]. In alignment with these findings, the present study evaluated the structure of mandibular trabecular bone in individuals using antiresorptive drugs by analyzing FD values across three distinct ROIs.

Digital panoramic radiography is a widely used, cost-effective, and easily accessible imaging modality for detecting changes in bone mineral density. It is considered to be a reliable tool for assessing trabecular and cortical bone and is frequently utilized in dental practice [17,23,24]. In this study, FD-based measurements were conducted using diagnostic panoramic radiographs, and no additional radiographic imaging was performed for the included patients. The ICCs were remarkably high for all ROI measurements, with the condyle and angle regions demonstrating an excellent agreement. These findings underscore the high consistency and reliability of the repeated measurements, reinforcing the robustness of the applied FD analysis approach.

Previous studies evaluating FD values in patients using antiresorptive drugs have reported various findings based on different imaging modalities and analysis regions. Demiralp et al. [37] compared FD values obtained from panoramic radiographs of oncologic patients receiving bisphosphonates with those of 33 healthy individuals. Although the FD values were higher in the study group than in the control group, the difference was not statistically significant. Additionally, the authors identified the area superior to the mandibular canal and distal to the second premolar as the most suitable region for FD analysis. Similarly, Barngkgei et al. [24] conducted an FD analysis on selected ROIs in panoramic radiographs of patients undergoing bisphosphonate therapy for osteoporosis for 4.5 to 5 years and observed no statistically significant change in FD values. Sindeaux et al. [38] also reported that trabecular bone FD values were lower in osteoporotic individuals than in healthy individuals, but this difference was not statistically significant. Torres et al. [36] investigated patients with BRONJ using cone-beam computed tomography (CBCT) and compared them with healthy individuals. They reported that the FD values were higher in the study group than in the control group. However, this difference was statistically significant only for the trabecular bone superior to the mandibular canal. Moreover, the authors emphasized that bisphosphonate-related bone changes were most commonly observed in the alveolar bone and suggested that regions adjacent to the alveolar bone might be the most suitable areas for detecting jaw osteonecrosis.

In the current study, a comparison of FD values between osteoporosis and oncologic patients revealed no statistically significant differences in the mandibular condyle and mandibular angle regions. However, the mean FD values in the molar region of osteoporosis patients (1.26 ± 0.18) were significantly lower than those of the control group (1.32 ± 0.07). Additionally, although the mean FD values in the mandibular angle and molar regions of the osteoporosis group were lower than those of the oncologic patients, this difference was not statistically significant. Among the analyzed regions, the molar region demonstrated the most pronounced changes in FD measurements, aligning with the findings of Torres et al. [36].

However, methodological differences among studies complicate direct comparisons between findings. While Torres et al. [36] conducted their study using CBCT, the present study utilized panoramic radiographs. FD analysis is considered reliable only when performed on radiographs with the same spatial resolution. Although CBCT provides a higher resolution, it was not chosen in this study due to its limited use in routine clinical practice and the higher radiation dose required. Differences between studies may also stem from variations in patient populations, ROI selection, treatment duration, and dosage. Additionally, local factors influencing FD measurements, particularly occlusal forces and the presence or absence of teeth, may alter trabecular bone density outcomes [24].

FD analysis studies evaluating changes in the trabecular bone structure of patients using bisphosphonates have previously examined the effects of age and gender on FD values from various perspectives. Demiralp et al. [37] compared the FD values obtained from panoramic radiographs of oncologic patients using bisphosphonates with those of a control group consisting of 33 healthy individuals. Their study found no significant correlation between age and FD values; however, male patients exhibited significantly higher FD values than female patients.

Similarly, Geçkil et al. [39] evaluated FD, panoramic mandibular index (PMI), and mandibular cortical width (MCW) measurements in panoramic radiographs of 30 patients using bisphosphonates and a control group of 30 healthy individuals. They reported no significant association between age and FD values. However, male participants demonstrated significantly higher values in ROI-3 (anterior to the mental foramen region), PMI, and MCW than females.

Kurşun et al. [23] aimed to assess changes in mandibular trabecular bone using FD analysis on 100 patients using bisphosphonates and 100 healthy individuals, categorized according to the type of prosthesis used (completely removable, partially removable, or fixed prostheses). Their findings revealed that the FD values in the region superior to the mandibular canal, distal to the second premolar, were significantly higher in male patients than in female ones.

In the present study, the effects of age and gender on FD values were analyzed in a cohort of patients using antiresorptive drugs and a healthy control group. Our findings indicated no statistically significant correlation between age and FD values across the ROIs in oncologic patients and the control group. However, in the osteoporosis group, a significant negative correlation was observed between age and the FD values in the mandibular angle region. In contrast, no significant correlation was found in the mandibular condyle and molar regions in osteoporotic patients. When all participants were analyzed collectively, a significant negative correlation was detected between age and the FD values in the mandibular angle region, suggesting that the FD values in this area decrease with an advancing age. 

Unlike in previous studies, no statistically significant differences were found in the FD values across ROIs between male and female participants in the oncologic patient, osteoporosis, and control groups. This discrepancy may be attributed to the non-homogeneous gender distribution in the osteoporosis group. Additionally, the limited sample size might have reduced sensitivity to gender-related differences.

Geçkil et al. [39] evaluated trabecular bone FD values in patients using bisphosphonates and compared them with those of healthy individuals. They assessed the effects of oral and IV bisphosphonate administration on jawbones but detected no significant differences between the selected ROIs. Similarly, Musulluoğlu et al. [17] investigated the impact of IV and oral bisphosphonate use on mandibular bone density in postmenopausal individuals. They reported that edentulous individuals receiving IV bisphosphonates exhibited higher FD values in the mandibular angle region than those using oral bisphosphonates. Additionally, in dentate individuals, IV administration was associated with higher FD values in the mandibular angle and condyle regions compared to oral administration. Furthermore, the FD values in the mandibular angle region were found to decrease as the duration of drug use increased.

In our study, the FD values in the mandibular condyle region were significantly higher in oncologic patients receiving denosumab than those receiving IV zoledronic acid. However, no statistically significant differences were observed in the mandibular angle and molar regions based on drug type. In the osteoporosis group, no significant differences were detected between individuals using IV zoledronic acid and those using oral alendronic acid in the mandibular condyle, angle, or molar regions.

When evaluating the impact of treatment duration, oncologic patients who had been using antiresorptive drugs for 6 years or more exhibited significantly higher FD values in the mandibular condyle region than those who had been using them for 1–5 years. However, no significant differences were observed in the mandibular angle and molar regions based on treatment duration. In the osteoporosis group, no statistically significant differences were found between individuals using antiresorptive drugs for 1–5 years and those using them for 6 years or more in any of the ROIs.

The differences between our study and the existing literature may be attributed to variations in imaging modalities, patient population characteristics, treatment duration, dosage, and drug types. Unlike previous studies, our study included individuals using antiresorptive drugs for osteoporosis as well as oncological reasons. Furthermore, this study distinguishes itself from prior research by evaluating the effects of denosumab in addition to bisphosphonates. However, differences between dentate and edentulous patients were not considered, which may have influenced the findings. Previous studies have reported a lower bone density in edentulous regions [17,24], which may have contributed to the discrepancies between them and our study findings.

The majority of previous studies have utilized FD analysis, the PMI, the mandibular cortical index (MCI), and MCW measurements to assess cortical and trabecular bone changes in patients using bisphosphonates [40,41]. Furthermore, existing research has predominantly focused on patients receiving bisphosphonates, while FD analysis data from individuals using denosumab remain limited.

This study is among the first to comprehensively evaluate the effects of antiresorptive therapy on bone structure in both osteoporosis and oncology patients using panoramic radiographs. By incorporating FD analysis, it assessed the impacts of different drug types while considering key factors such as administration route, treatment duration, patient age, and gender. This integrated approach provides a deeper understanding of antiresorptive therapies’ effects on bone and helps to address existing gaps in the literature.

The limitations of this study include its relatively small sample size, which may limit its statistical power to detect significant associations between certain variables. Accordingly, the results should be interpreted as preliminary data, requiring confirmation through validation studies with larger cohorts. The study’s retrospective design also limits the possibility of establishing causal relationships. Moreover, as FD analyses were conducted using panoramic radiographs, direct comparisons with previous studies employing different imaging methods were not feasible. A balanced gender distribution was not achieved in the osteoporosis group, which included only one male patient. In contrast, the oncologic patient group was matched with the control group in terms of age and gender. This imbalance may limit the interpretability of the FD measurements in the osteoporosis group. Although patients using systemic corticosteroids were excluded, the presence of other systemic diseases and vitamin D levels—which can influence bone metabolism— were not assessed. Given these limitations, future research should focus on prospective studies with larger sample sizes and incorporate comparisons of different imaging modalities to further elucidate the effects of antiresorptive drugs on bone structure.

This study highlights the potential of FD analysis as a valuable clinical tool for evaluating bone structural changes in patients undergoing antiresorptive therapy. FD analysis of digital panoramic radiographs enables the quantitative assessment of trabecular bone microarchitecture, facilitating the identification of medication-induced skeletal alterations. As a cost-effective, accessible, and clinically applicable method, it is a promising tool for monitoring bone structure in patients receiving bisphosphonates or denosumab. However, although FD analysis holds significant clinical potential, further research is required to fully establish its clinical relevance in detecting bone changes and to clarify its role in the clinical decision-making process.

Taken together, the results of this study demonstrate the potential of FD analysis as a promising, cost-effective, and accessible tool for assessing trabecular bone changes in dentistry. However, considering its limitations, further prospective studies with larger cohorts are essential to better understand the impact of antiresorptive therapies on trabecular bone structure.

## 5. Conclusions

This study evaluated the effects of antiresorptive therapy on mandibular trabecular bone structure using an FD analysis of panoramic radiographs and demonstrated its potential as a non-invasive tool for assessing bone changes in patients undergoing antiresorptive treatment. Our findings indicated that denosumab and bisphosphonates influenced trabecular bone differently, with denosumab being associated with higher FD values in the condyle region. Additionally, long-term antiresorptive therapy (≥ 6 years) was linked to increased FD values in specific regions, while osteoporosis patients exhibited lower FD values in the molar region than controls. The age-related decline in FD values in the mandibular angle further underscored the role of bone turnover in long-term remodeling. These results support the clinical relevance of FD analysis for evaluating drug-induced skeletal changes. However, further prospective studies are needed to validate these findings and refine their clinical application. 

## Figures and Tables

**Table 1:** Demographic and clinical characteristics of patients receiving antiresorptive therapy.

	Osteoporosis Group (*n* = 23)	Oncologic Patient Group (*n* = 25)	Control Group (*n* = 25)
Age (Mean age ± standard deviation)	68.78 ± 9.6	59.16 ± 11.32	59.16 ± 11.32
Gender			
Male	1 (3.7%)	13 (48.1%)	13 (48.1%)
Female	22 (47.8%)	12 (26.1%)	12 (26.1%)
Systemic Disease			
Breast cancer		8 cases (17%)	
Lung cancer		5 cases (10%)	
Prostate cancer		5 cases (10%)	
Multiple myeloma		2 cases (4%)	
Nasopharyngeal cancer		2 cases (4%)	
Ovarian cancer		1 case (2%)	
Liver cancer		1 case (2%)	
Renal cancer		1 case (2%)	
Osteoporosis	23 cases (49%)		
Drug Type	Zoledronic acid: 11 cases (23%) Alendronic acid: 12 cases (25%)	Zoledronic acid: 17 cases (35%) Denosumab: 8 cases (17%)	
Route of Drug Administration			
Intravenous	11 cases (23%)	17 cases (36%)	
Oral	12 cases (25%)		
Subcutaneous		8 cases (16%)	
Duration of Drug Therapy	1–5 years (27%) 6 years or more (21%)	1–5 years (40%) 6 years or more (12%)	

**Table 2:** Correlation between age and fractal dimension (FD) measurements in different mandibular regions.

c	Age	Condyle (ROI-1)	Angle (ROI-2)	Molar (ROI-3)
Oncologic Patient Group	r	−0.185	−0.097	−0.225
	*p*	0.375	0.644	0.279
Control Group	r	−0.189	−0.261	0.218
	*p*	0.365	0.207	0.296
Osteoporosis Group	r	−0.200	−0.557	0.101
	*p*	0.361	0.006 *	0.646

* *p* < 0.05 indicates statistical significance, r: Pearson and Spearman correlation coefficients.

**Table 3:** Comparison of FD measurements by gender.

			FD Measurement					
			Condyle (ROI-1)		Angle (ROI-2)		Molar (ROI-3)	
	**Gender**	***n***	M. (Min.–Max.)	Mean ± SD	M. (Min.–Max.)	Mean ± SD	M. (Min.–Max.)	Mean ± SD
Oncologic Patient Group	Female	12	1.29 (1.09–1.4)	1.26 ± 0.11	1.31 (0.99–1.37)	1.29 ± 0.1	1.33 (1.15–1.39)	1.3 ± 0.08
	Male	13	1.28 (1.1–1.4)	1.29 ± 0.09	1.32 (1.16–1.35)	1.29 ± 0.06	1.33 (1.21–1.4)	1.31 ± 0.06
Test Statistic			t = −0.825		Z = −0.435		t = −0.503	
*p*			0.418		0.663		0.620	
Control Group	Female	12	1.34 (1.11–1.38)	1.31 ± 0.08	1.28 (1.09–1.41)	1.29 ± 0.11	1.32 (1.23–1.39)	1.31 ± 0.06
	Male	13	1.28 (1.02–1.4)	1.28 ± 0.11	1.31 (1.11–1.4)	1.27 ± 0.1	1.35 (1.1–1.39)	1.33 ± 0.08
Test Statistic			Z = −0.381		Z = −0.653		Z = −1.251	
*p*			0.703		0.514		0.211	
Osteoporosis Group	Female	22	1.31 (1.1–1.4)	1.29 ± 0.07	1.27 (1.16–1.41)	1.27 ± 0.06	1.24 (1.06–1.97)	1.27 ± 0.18
	Male	1	1.26 (1.26–1.26)	1.26 ± 0	1.27 (1.27–1.27)	1.27 ± 0	1.08 (1.08–1.08)	1.08 ± 0

t: independent samples t-test statistic, Z: Mann–Whitney U test statistic, M: mean, FD: fractal di-mension, SD: standard deviation, Min: minimum, Max: maximum.

**Table 4:** Comparison of FD measurements among study and control groups.

	Oncologic Patient Group ^1^		Control Group ^2^		Osteoporosis Group ^3^		Test Statistic	*p*	Bonferroni (*p*)
	**M. (Min.–Max.)**	**Mean** ±**SD**	**M. (Min.–Max.)**	Mean ± SD	M. (Min.–Max.)	Mean ± SD			
Condyle (ROI-1)	1.28 (1.09–1.4)	1.27 ± 0.1	1.32 (1.02–1.4)	1.29 ± 0.1	1.3 (1.1–1.4)	1.29 ± 0.07	X^2^ = 0.556	0.757	-
Angle (ROI-2)	1.32 (0.99–1.37)	1.29 ± 0.08	1.31 (1.09–1.41)	1.28 ± 0.1	1.27 (1.16–1.41)	1.27 ± 0.06	X^2^ = 2.846	0.241	-
Molar (ROI-3)	1.33 (1.15–1.4)	1.3 ± 0.07	1.34 (1.1–1.39)	1.32 ± 0.07	1.23 (1.06–1.97)	1.26 ± 0.18	X^2^ = 10.063	0.007 *	3 < 2

* X^2^: Kruskal–Wallis test statistic, *p* < 0.05 indicates statistical significance, Bonferroni correction: significant difference observed between osteoporosis (Group 3) and control (Group 2) in the molar region (3 < 2), M: mean, SD: standard deviation, Min: minimum, Max: maximum. ^1^ Oncologic Patient Group: Patients diagnosed with oncologic conditions. ^2^ Control Group: Healthy individuals without any known systemic bone-related diseases. ^3^ Osteoporosis Group: Patients diagnosed with osteoporosis.

**Table 5:** Comparison of FD measurements by antiresorptive drug type and administration route.

			FD Measurement					
			Condyle (ROI-1)		Angle (ROI-2)		Molar (ROI-3)	
	**Medication**	***n***	M. (Min.–Max.)	Mean ± SD	M. (Min.–Max.)	Mean ± SD	M. (Min.–Max.)	Mean ± SD
Oncologic Patient Group	Denosumab	8	1.35 (1.25–1.4)	1.34 ± 0.04	1.31 (1.19–1.34)	1.29 ± 0.05	1.34 (1.15–1.39)	1.31 ± 0.07
	IV Zoledronic Acid	17	1.23 (1.09–1.4)	1.24 ± 0.1	1.32 (0.99–1.37)	1.29 ± 0.09	1.31 (1.19–1.4)	1.3 ± 0.06
Test Statistic			t = 2.791		Z = −0.699		Z = −0.466	
*p*			0.010 *		0.485		0.641	
Osteoporosis Group	IV Zoledronic Acid	11	1.32 (1.1–1.4)	1.29 ± 0.09	1.27 (1.19–1.32)	1.26 ± 0.04	1.31 (1.12–1.41)	1.27 ± 0.1
	Oral Alendronic Acid	12	1.3 (1.22–1.36)	1.29 ± 0.05	1.27 (1.16–1.41)	1.27 ± 0.07	1.18 (1.06–1.97)	1.25 ± 0.24
Test Statistic			t = 0.023		t = −0.698		Z = −1.477	
*p*			0.982		0.493		0.140	

* *p* < 0.05 indicates statistical significance, t: independent samples t-test statistic, Z: Mann–Whitney U test statistic, IV: intravenous, M: mean, FD: fractal dimension, SD: standard deviation, Min: minimum, Max: maximum.

**Table 6:** Comparison of FD measurements by antiresorptive treatment duration.

			FD Measurement					
			Condyle (ROI-1)		Angle (ROI-2)		Molar (ROI-3)	
	**Medication Duration (Years)**	***n***	M. (Min.–Max.)	Mean ± SD	M. (Min.–Max.)	Mean ± SD	M. (Min.–Max.)	Mean ± SD
Oncologic Patient Group	1–5 years	19	1.27 (1.09–1.38)	1.25 ± 0.09	1.31 (0.99–1.37)	1.28 ± 0.09	1.32 (1.15–1.37)	1.29 ± 0.06
	6 years or more	6	1.36 (1.19–1.4)	1.34 ± 0.08	1.34 (1.25–1.36)	1.33 ± 0.04	1.36 (1.23–1.4)	1.34 ± 0.07
Test Statistic			Z = −1.972		Z = −1.654		Z = −1.654	
*p*			0.049 *		0.098		0.098	
Osteoporosis Group	1–5 years	13	1.32 (1.1–1.4)	1.3 ± 0.08	1.27 (1.19–1.41)	1.27 ± 0.06	1.29 (1.14–1.41)	1.26 ± 0.09
	6 years or more	10	1.28 (1.18–1.36)	1.28 ± 0.06	1.26 (1.16–1.33)	1.26 ± 0.06	1.18 (1.06–1.97)	1.26 ± 0.27
Test Statistic			t = 0.602		t = −1.339		Z = −1.240	
*p*			0.554		0.194		0.215	

* *p* < 0.05 indicates statistical significance, t: independent samples t-test statistic, Z: Mann–Whitney U test statistic, M: mean, FD: fractal dimension, SD: standard deviation, Min: minimum, Max: maximum.

## Data Availability

The data presented in this study are available on request from the corresponding author due to privacy and ethical restrictions, as the dataset contains patient information that cannot be shared publicly.

## Abbreviations

The following abbreviations are used in this manuscript:

FD	Fractal Dimension
IV	Intravenous
BRONJ	Bisphosphonate-Related Osteonecrosis of the Jaw
MRONJ	Medication-Related Osteonecrosis of the Jaw
AAOMS	American Association of Oral and Maxillofacial Surgeons
TIFF	Tagged Image File Format
ROIs	Regions of Interest
ICC	Intraclass Correlation Coefficient
CBCT	Cone-Beam Computed Tomography
PMI	Panoramic Mandibular Index
MCW	Mandibular Cortical Width
MCI	Mandibular Cortical Index
